# The association between olfactory and gustatory dysfunction and chronic kidney disease

**DOI:** 10.1186/s12882-021-02659-6

**Published:** 2022-01-18

**Authors:** Api Chewcharat, Elizabeth A. Phipps, Khushboo Bhatia, Sahir Kalim, Andrew S. Allegretti, Meghan E. Sise, Teodor G. Păunescu, Rituvanthikaa Seethapathy, Sagar U. Nigwekar

**Affiliations:** 1 Department of Medicine, Mount Auburn Hospital, Harvard Medical School, 330 Mount Auburn Street, Cambridge, MA 02138 USA; 2 Division of Nephrology, Department of Medicine, Massachusetts General Hospital, Harvard Medical School, Boston, MA USA

## Abstract

**Background:**

Olfactory and gustatory changes may contribute to poor appetite and food aversion in chronic kidney disease (CKD), though the prevalence of olfactory and gustatory dysfunction is not known in the CKD population.

**Methods:**

We conducted a cross-sectional study among 3527 US adults aged ≥40 years old in the National Health and Nutrition Examination Survey (NHANES) between 2013 and 2014. We measured the prevalence of olfactory and gustatory dysfunction among patients with CKD defined as eGFR < 60 ml/min/1.73m^2^ using the “scratch and sniff” NHANES Pocket Smell Test and quinine whole-mouth test. We also examined the association between CKD and olfactory/gustatory dysfunction, and nutritional markers.

**Results:**

The prevalence of olfactory dysfunction was 30% among CKD and 15% among non-CKD (*p* < 0.001). The prevalence of gustatory dysfunction was 13% among CKD and 17% among non-CKD (*p* = 0.10). After adjusting for confounders, CKD was significantly associated with olfactory dysfunction (OR = 1.47, 95% CI [1.07, 2.01]; *p* = 0.02) but not gustatory dysfunction (OR = 1.76, 95%CI [0.99, 3.11]; *p* = 0.05). Among the CKD population, the odds of olfactory dysfunction was 72% higher for every 10 kg decrease in grip strength (OR = 1.72, 95% CI [1.39, 2.13]; adjusted *p* = 0.005).

**Conclusion:**

CKD was associated with higher odds of olfactory but not gustatory dysfunction. Olfactory dysfunction was associated with lower grip strength among those with CKD. Screening and early intervening on olfactory dysfunction among CKD may preserve muscle strength and improve nutritional status in this vulnerable population.

**Supplementary Information:**

The online version contains supplementary material available at 10.1186/s12882-021-02659-6.

## Introduction

Olfactory and gustatory dysfunction are common in the general population, especially among the elderly, but are rarely recognized and addressed [1]. Previous analyses of National Health and Nutrition Examination Survey (NHANES) have reported the prevalence of olfactory dysfunction was 14% and gustatory dysfunction was 18% among general population [2]. Common risk factors include age, diabetes mellitus, neurodegenerative diseases, cancer, medications and trauma [3]. Olfactory and gustatory dysfunction have been shown to be associated with anorexia and food aversion. These symptoms lead to poor nutritional status, fatigue, weakness, and malnutrition which contribute to increased morbidity and mortality [1, 4–6].

Decreased appetite and food aversion are frequently seen in patients with chronic kidney disease (CKD) though mechanisms remain unclear [7, 8]. Previous small studies have reported manifestations of olfactory and gustatory dysfunction among the CKD population [9, 10]. High levels of urea, trimethylamine and lower serum zinc as a result of CKD may lead to alteration in olfactory and gustatory function [9, 11]. Nevertheless, the prevalence of olfactory and gustatory dysfunction among patients with CKD has not been defined in large nationally representative cohorts. Previous study showed that simple intervention such as nasal theophylline may alleviate olfactory dysfunction among patients with CKD [10]. Therefore, early screening for olfactory and gustatory dysfunction among CKD patients may provide a potential opportunity to prevent malnutrition.

We sought to quantify the extent that CKD is associated with olfactory and gustatory dysfunction relative to the general population, explore the role of zinc on these dysfunctions and how these dysfunctions might contribute towards markers of malnutrition.

## Material and methods

### Data source and study population

NHANES is an ongoing survey study of both children’s and adults’ health and nutritional status in the United States conducted by the US Centers for Disease Control and Prevention with the goal of monitoring health status of the US general population. NHANES has been conducted in two-year cycles since 1999. Our study conducted a cross-sectional study in the NHANES 2013–2014 which included all 3708 US males and females aged ≥40 years old who enrolled in smell and taste examination. Participants were excluded if they were pregnant or breastfeeding. This study was approved by the Mount Auburn Hospital/BIDMC institutional review board; the need for informed consent was waived due to the publicly available nature of the de-identified datasets in NHANES.

### Assessment of smell and taste

In 2013–2014, NHANES examined the olfactory and gustatory dysfunction among participants aged ≥40 years old. Participants were excluded if they were pregnant or breastfeeding. The smell testing was a brief 8-item “scratch and sniff” NHANES Pocket Smell Test, manufactured by Sensonics International, Haddon Heights, New Jersey, USA. There were eight odorants comprised of chocolate, strawberry, smoke, leather, soap, grape, onion and natural gas presenting in a fixed order. These stimuli were released by using a plastic stylus to scratch the odor test strips in a standardized manner. Then participants were required to identify each odorant from four choices. These eight odorants are part of the 40-item University of Pennsylvania Smell Identification Test (UPSIT) [12]. Olfactory dysfunction was defined as incorrectly identifying three or more of the eight odors. This definition corresponds to being unable to correctly identify 29 or more of the 40 odors using UPSIT test [2]. NHANES smell test demonstrated moderate-to-good test-retest reliability in a recent validation study [13].

For taste assessment, the examinations included a tongue-tip taste test and whole-mouth taste test. Participants who were unable to grade the three light intensity standards on the generalized labeled magnitude scale (gLMS) [14, 15] or allergic to quinine were excluded from participating in taste test. Two tastants including 0.32 mg of quinine for bitter taste and 58.5 mg/ml of sodium chloride (NaCl) for salty taste were applied to the tip of the tongue by using a cotton swab applicator in a fixed presentation order. The mouth was rinsed with water and waited for 30 s before applying the next tastant. Then participants were asked to identify the taste and grade the perceived intensity on the gLMS. After completing tongue tip taste testing, participants proceeded with whole mouth taste test. Three tastants including 10 ml of 19.5 and 58.5 mg/ml of NaCl and 0.32 mg/ml of quinine were swished for 3 s and spitted out. The mouth was then rinsed with water using the same step as tongue tip taste testing. Gustatory impairment was defined as failing to identify quinine in the whole-mouth test. As quinine whole-mouth taste testing has been demonstrated to be a reasonable tool for overall taste functioning as described by previous studies [2, 13].

### Kidney impairment

Estimated glomerular filtration rate (eGFR) was calculated using the Chronic Kidney Disease Epidemiology Collaboration equation (CKD-EPI). CKD was defined as eGFR < 60 ml/min/1.73 m^2^. In secondary analysis, we used the expanded definition of CKD to include eGFR < 60 ml/min/1.73 m^2^ or urine albumin creatinine ratio ≥ 30 mg/g. These definitions have been used in NHANES analyses [16, 17].

### Sociodemographic characteristics and other variables

Covariates of interest included age, sex, race (non-Hispanic white, non-Hispanic black, Hispanic and other race/multiracial), educational attainment (below high school, high school and some college or above), marital status (married or cohabiting and not married or cohabiting), family income to poverty ratio (low defined as ratio < 1.3, middle defined as 1.3–3.5 and high defined as > 3.5), alcohol drinking (non-drinker, 1–3 drinks/week and ≥ 4 drinks/week), cigarette smoking status (never, past and current smoker), diabetes, hypertension, obesity, history of cardiovascular disease, history of cancer and depression. Further information was described in Supplementary Methods.

### Serum zinc

Serum zinc was collected in one-third of participants in NHANES cycle 2013–2014. Inductively coupled plasma dynamic reaction cell mass spectrometry (ICP-DRC-MS) was used to quantify the level of zinc.

### Nutritional status

Nutritional status in our study was assessed using total serum cholesterol, LDL cholesterol, albumin, grip strength and protein-energy malnutrition; these have been assessed CKD populations in previous studies [10, 18–20]. Briefly, grip strength was measured by a Takei Digital Grip Strength handgrip dynamometer (Model T.K.K.5401). The grip size of the dynamometer was adjusted according to the participant’s hand size. After practicing, the participant was asked to squeeze the dynamometer as hard as possible with each hand for three attempts. The sum of the largest reading from each hand was used to represent the combined grip strength [21]. Protein-energy malnutrition in our study was defined by the presence of at least 3 from 5 of the following criteria: 1) serum albumin ≤3.7 g/dl, 2) weight ≤ 63.9 kg in male and ≤ 51.8 kg in female, 3) total serum cholesterol < 159 mg/dl, 4) reported total energy intake < 15 kcal/kg/day and 5) protein intake < 0.5 g/kg/day [18].

### Statistical analysis

The prevalence of olfactory and gustatory dysfunction among participants with and without CKD was calculated by incorporating analytic survey weights and design factors to account for the unequal probabilities of selection, oversampling, and nonresponse. Baseline characteristics were summarized and compared between participants with and without olfactory dysfunction among those with and without CKD using Wald F-test for continuous variables or chi-square tests for categorical variables.

Multivariable logistic regression accounting for survey weights was utilized to assess the association between CKD (main predictor) and olfactory (binary outcome) and gustatory dysfunction (binary outcome). We adjusted for potential confounders specified a priori including age, sex, race, educational attainment, marital status, family income to poverty ratio, alcohol drinking, cigarette smoking status, diabetes, hypertension, obesity, history of cardiovascular disease, history of cancer and depression. To evaluate the association between olfactory and gustatory dysfunction and nutritional markers among participants with impaired kidney function, multivariable linear regression and multivariable logistic regression accounting for survey weights were used. Sensitivity analysis was performed by using expanded definition of CKD to include both eGFR < 60 ml/min/1.73 m^2^ or urine albumin creatinine ratio ≥ 30 mg/g.

Data on income ratio were missing for 283 (8%), and data of alcohol consumption were missing for 750 (21%). Hence, we used Markov chain Monte Carlo multiple imputations to simulate five complete datasets. All analyses were performed in each dataset and averaged utilizing “mi estimate” combined with “svy” commands in STATA 14.1 (StataCorp LLC, Texas, USA). Imputed and observed estimates were compared to evaluate for the reasonableness of the imputation model. Results were considered statistically significant if a two-sided α < 0.05 threshold was reached. Sidak-Holm adjusted *p*-values were calculated for the analyses for the association between olfactory and gustatory dysfunction, and nutritional markers due to potential type 1 error from multiple comparisons.

## Results

**Fig. 1:**
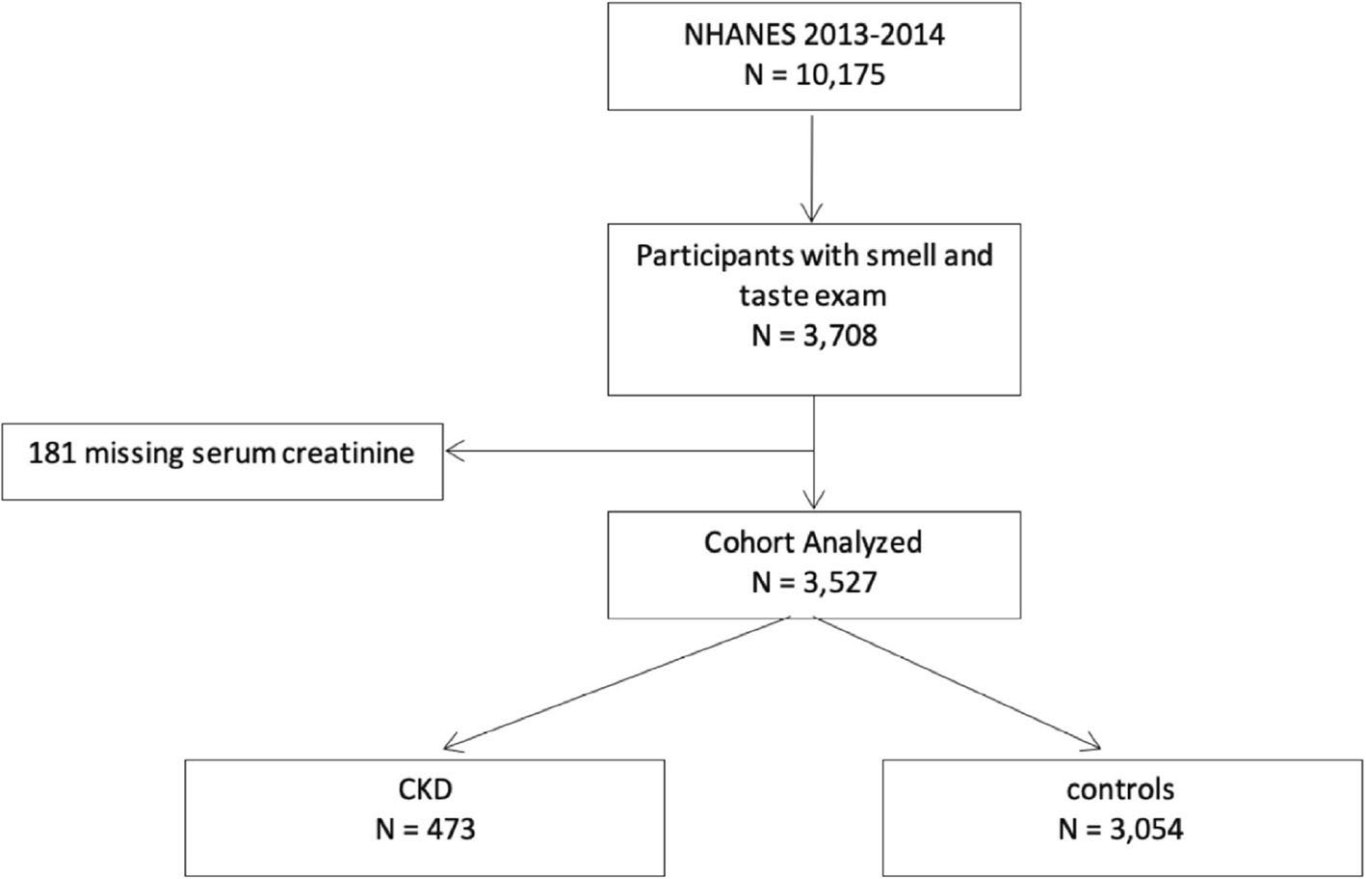
Flow chart of included population

A total of 3527 participants were included in this analysis, 473 participants had CKD, and 3054 participants were included as controls defined as participants without CKD (Fig. 1).

The prevalence of olfactory dysfunction was higher among participants with CKD compared to controls, 30% vs. 15% (*p* < 0.001). In contrast, the prevalence of gustatory dysfunction was not significantly different between CKD and controls, 13% vs. 17% (*p* = 0.10). Demographic and clinical characteristics of participants included in our study are shown in Table 1. Among participants with CKD, those with olfactory dysfunction had lower eGFR, were older, more likely to be Black or Hispanic, less educated, had lower family income to poverty ratio, had less alcohol consumption, and had higher cardiovascular disease. Among controls, those with olfactory dysfunction were older, less likely to be more likely to be Black or Hispanic, less educated, had lower family income to poverty ratio, had less alcohol consumption, and had higher incidence of hypertension, cardiovascular disease and cancer.

**Table 1:** Demographic and Clinical Characteristics of Participants from NHANES, 2013 to 2014

Characteristics	CKD			Controls (without CKD)		
	Normal olfactory function (*n* = 292)	With olfactory dysfunction (*n* = 181)	*P*-value	Normal olfactory function (*n* = 2471)	With olfactory dysfunction (*n* = 583)	*P*-value
Gustatory dysfunction, %	8.4	16.0	0.07	17.9	20.0	0.37
Estimated glomerular filtration rate (eGFR), ml/min/1.73 m2	48.1 (0.9)	43.9 (1.4)	0.03	88.8 (0.4)	87.0 (1.0)	0.09
Age, y	67.6 (0.9)	73.7 (1.0)	0.002	55.5 (0.2)	60.9 (0.9)	< 0.001
Male, %	43.0	45.2	0.60	47.0	54.2	0.07
Race, %			0.01			0.003
- Non-Hispanic white	83.7	75.7		72.5	62.2	
- Non-Hispanic black	7.5	12.3		9.4	11.9	
- Hispanic	4.7	8.5		11.3	15.1	
- Other	4.2	3.5		6.8	10.9	
Education attainment, %			< 0.001			< 0.001
- Less than high school	12.3	31.5		13.1	25.3	
- High school	22.2	21.8		21.8	20.4	
- Above high school	65.5	45.7		65.2	54.2	
Marital status, %			0.006			0.67
- Married or cohabiting	39.9	54.9		68.9	67.1	
- Not married or cohabiting	60.1	45.1		31.1	32.9	
Family income to poverty ratio, %			0.002			0.001
- < 1.3	21.8	28.3		19.1	28.1	
- 1.3–3.5	35.0	53.4		32.7	42.0	
- > 3.5	43.3	18.4		48.2	29.9	
Tobacco use, %			0.30			0.07
- Never	50.5	51.1		55.3	52.3	
- Past	40.0	34.2		26.1	32.6	
- Current	9.5	14.0		18.6	15.0	
Alcohol drinking			0.05			0.002
- Non-drinker	36.0	52.5		22.7	37.9	
- 1–3 drinks	45.6	35.7		47.3	36.7	
- > 4 drinks	18.4	11.8		29.9	25.5	
Obesity, %	43.1	35.9	0.28	39.4	34.0	0.06
Underlying disease						
Chronic hypertension, %	82.1	84.0	0.73	53.2	60.9	0.007
Cardiovascular disease, %	25.5	38.2	0.009	7.1	10.1	0.04
Diabetes, %	32.3	32.8	0.93	12.4	15.4	0.09
Depression, %	30.4	32.7	0.76	23.3	20.7	0.30
Cancer, %	26.1	27.0	0.86	13.2	18.9	0.02

In our study, there were 289 participants (61%) with CKD stage 3a defined as eGFR 45–59 ml/min/1.73m^2^, 129 participants (27%) with CKD stage 3b defined as eGFR 30–44 ml/min/1.73m^2^, 34 participants (7%) with CKD stage 4 defined as eGFR 15–29 ml/min/1.73m^2^ and 21 participants (5%) with CKD stage 5 defined as eGFR < 15 ml/min/1.73m^2^. Across stages of CKD, the weighted prevalence of olfactory dysfunction was 25.5% among stage 3a, 39.8% among stage 3b, 34.4% among stage 4 and 51.2% among stage 5. There was a significant increasing trend of the prevalence of olfactory dysfunction across CKD stage 3a-5 (p for trend = 0.03). The prevalence of gustatory dysfunction was 10.9% among stage 3a, 8.2% among stage 3b, 4.9% among stage 4 and 10.1% among stage 5 without a significant trend (p for trend = 0.42) as shown in Table 2.

**Table 2:** Stages of kidney impairment and weighted prevalence of olfactory and gustatory dysfunction

Stages of kidney impairment	Prevalence of olfactory dysfunction	Prevalence of gustatory dysfunction
eGFR 45–59 ml/min/1.73 m^2^	25.5% (*n* = 100)	10.9% (*n* = 31)
eGFR 30–44 ml/min/1.73 m^2^	39.8% (*n* = 58)	8.2% (*n* = 11)
eGFR 15–29 ml/min/1.73 m^2^	34.4% (*n* = 12)	4.9% (*n* = 2)
eGFR < 15 ml/min/1.73 m^2^	51.2% (*n* = 11)	10.1% (*n* = 2)
	*P* for trend = 0.03	*P* for trend = 0.42

After adjusting for age, sex, race, educational attainment, marital status, family income to poverty ratio, alcohol drinking, cigarette smoking status, diabetes, hypertension, obesity, history of cardiovascular disease, history of cancer and depression, the odds of having olfactory dysfunction among participants with CKD was 47% higher than those without CKD (OR = 1.47, 95%CI [1.07, 2.01]; *p* = 0.02). Regarding gustatory dysfunction, after adjusting for potential confounders, there was no significant association between CKD and the odds of having gustatory dysfunction (OR = 1.76, 95%CI [0.99, 3.11]; *p* = 0.05) (Table 3). These findings were consistent in a sensitivity analysis using expanded definition of CKD (Supplementary Table 1).

**Table 3:** The association between impaired kidney function and olfactory and gustatory dysfunction

	Odds ratio of having olfactory dysfunction				Odds ratio of having gustatory dysfunction			
	Crude	*p*-value	Adjusted^a^	*p*-value	Crude	*p*-value	Adjusted^a^	*p*-value
CKD (eGFR< 60 ml/min/1.73 m^2^)	2.61 (2.06, 3.31)	< 0.001	1.47 (1.07, 2.01)	0.02	0.61 (0.34, 1.10)	0.10	1.76 (0.99, 3.11)	0.05

^a^Multivariable logistic regression model was adjusted for age, sex, race, educational attainment, marital status, family income to poverty ratio, alcohol drinking, cigarette smoking status, diabetes, hypertension, obesity, history of cardiovascular disease, history of cancer and depression

In terms of serum zinc, data were available among 137 (29%) of CKD participants and 1001 (33%) controls. There was no significant difference in serum zinc between participants with CKD and controls, 79.2 μg/dL vs 81.6 μg/dL (*p*-value = 0.15). Among CKD participants, there was no significant association between serum zinc and odds of olfactory dysfunction in both unadjusted model (OR = 1.17 for every 10 μg/dL decrease in serum zinc, 95% CI [0.79, 1.60]; adjusted *p*-value = 0.50) and adjusted model (OR = 1.02 for every 10 μg/dL decrease in serum zinc, 95% CI [0.83, 1.25]; adjusted *p*-value = 0.83). Similarly, there was no significant association between serum zinc and odds of olfactory dysfunction among controls in either unadjusted model (OR = 0.94 for every 10 μg/dL decrease in serum zinc, 95% CI [0.79, 1.13]; adjusted *p*-value = 0.52) or adjusted model (OR = 1.05 for every 10 μg/dL decrease in serum zinc, 95% CI [0.90, 1.23]; adjusted *p*-value = 0.50). For gustatory dysfunction, among participants with CKD, there was no significant association between serum zinc and odds of gustatory dysfunction in either unadjusted model (OR = 1.27 for every 10 μg/dL decrease in serum zinc, 95% CI [0.94, 1.61]; adjusted *p*-value = 0.06) or adjusted model (OR = 1.24 for every 10 μg/dL decrease in serum zinc, 95% CI [0.81, 1.90]; adjusted p-value = 0.30). Comparably, there was no significant association between serum zinc and odds of gustatory dysfunction among controls in both unadjusted model (OR = 1.03 for every 10 μg/dL decrease in serum zinc, 95% CI [0.92, 1.15]; adjusted *p*-value = 0.61) and adjusted model (OR = 1.04 for every 10 μg/dL decrease in serum zinc, 95% CI [0.89, 1.22]; adjusted p-value = 0.60) (Table 4).

**Table 4:** The association between serum zinc and olfactory and gustatory dysfunction

	Odds ratio of having olfactory dysfunction per 10 μg/dL decrease in serum zinc				Odds ratio of having gustatory dysfunction per 10 μg/dL decrease in serum zinc			
	Crude	*p*-value	Adjusted^a^	*p*-value	Crude	*p*-value	Adjusted^a^	*p*-value
CKD (eGFR< 60 ml/min/1.73 m^2^)	1.17 (0.79, 1.60)	0.50	1.02 (0.83, 1.25)	0.83	1.27 (0.94, 1.61)	0.06	1.24 (0.81, 1.90)	0.30
Controls (without CKD)	0.94 (0.79, 1.13)	0.52	1.05 (0.90, 1.23)	0.50	1.03 (0.92, 1.15)	0.61	1.04 (0.89, 1.22)	0.60

^a^Multivariable logistic regression model was adjusted for age, sex, race, educational attainment, marital status, family income to poverty ratio, alcohol drinking, cigarette smoking status, diabetes, hypertension, obesity, history of cardiovascular disease, history of cancer and depression

In univariate analysis, the odds of having olfactory dysfunction was 5% higher for every 10 mg/dl decrease in total cholesterol (OR 1.05, 95% CI [1.02, 1.08]; adjusted *p* = 0.004), 16% higher for every 10 kg decrease in grip strength (OR 1.16, 95% CI [1.09, 1.23]; adjusted *p* = 0.004) and 100% higher for every 1 mg/dl decrease in serum albumin (OR 2.00, 95% CI [1.37, 2.94]; adjusted *p* = 0.02). After adjusting for age, sex, race, educational attainment, marital status, family income to poverty ratio, alcohol drinking, cigarette smoking status, diabetes, hypertension, obesity, history of cardiovascular disease, history of cancer and depression, the odds of having olfactory dysfunction was 25% higher for every 10 kg decrease in grip strength (OR 1.25, 95% CI [1.15, 1.37]; adjusted *p* = 0.006) (Table 5). In contrast, having gustatory dysfunction was not associated with any of the nutritional markers in both unadjusted and adjusted models as shown in Table 6. In patients with CKD, the odds of olfactory dysfunction was 37% higher for every 10 kg decrease in grip strength (OR 1.37, 95% CI [1.20, 1.56]; adjusted *p* = 0.004) and 144% higher for every 1 mg/dl decrease in serum albumin (OR 2.44, 95% CI [1.33, 4.54]; adjusted *p* = 0.02). However, after adjusting for potential confounders, the odds of olfactory dysfunction among CKD was 72% higher for every 10 kg decrease in grip strength (OR 1.72, 95% CI [1.39, 2.13]; adjusted *p* = 0.005) as shown in Table 5. On the contrary, gustatory dysfunction was not significantly associated with any of the nutritional markers in both unadjusted and adjusted models (Table 6). In sensitivity analysis among expanded CKD, there was no significant difference in any nutritional markers among those with and without olfactory dysfunction (Supplementary Table 2a) and gustatory dysfunction (Supplementary Table 2b).

**Table 5:** The association between olfactory dysfunction and nutritional markers

	Total cholesterol		LDL-cholesterol		Grip strength		Albumin		Protein-Energy malnutrition	
	Odds ratio	*p*-value^*^	Odds ratio	*p*-value^*^	Odds ratio	*p*-value^*^	Odds ratio	*p*-value^*^	Odds ratio	*p*-value^*^
Controls (without CKD)	Per 10 mg/dl decrease		Per 10 mg/dl decrease		Per 10 kg decrease		Per 1 mg/dl decrease			
Model 1	1.05 (1.02, 1.08)	0.004	1.06 (1.00, 1.11)	0.06	1.16 (1.09, 1.23)	0.004	2.00 (1.37, 2.94)	0.02	1.26(0.68, 2.34)	0.44
Model 2	1.02 (0.99, 1.05)	0.32	1.02 (0.97, 1.09)	0.60	1.25 (1.15, 1.37)	0.006	1.56 (0.89, 2.70)	0.60	1.01 0.50, 2.09)	0.98
CKD (eGFR< 60 ml/min/1.73 m^2^)										
Model 1	1.04(0.90, 1.10)	0.13	1.03 (0.87, 1.23)	0.62	1.37 (1.20, 1.56)	0.004	2.44 (1.33, 4.54)	0.02	0.81 (0.40, 1.63)	0.77
Model 2	1.02 (0.93, 1.11)	0.94	0.99 (0.83, 1.18)	0.95	1.72 (1.39, 2.13)	0.005	2.94 (1.39, 6.25)	0.08	0.88 (0.28, 2.76)	0.96

Model 1 was univariable model

Model 2 was adjusted for age, sex, race, educational attainment, marital status, family income to poverty ratio, alcohol drinking, cigarette smoking status, diabetes, hypertension, obesity, history of cardiovascular disease, history of cancer and depression

^*^p-value was calculated by Sidak-Holm technique to adjust for multiple comparisons

**Table 6:** The association between gustatory dysfunction and nutritional markers

	Total cholesterol		LDL-cholesterol		Grip strength		Albumin		Protein-Energy malnutrition	
	Odds ratio	*p*-value^*^	Odds ratio	*p*-value^*^	Odds ratio	*p*-value^*^	Odds ratio	*p*-value^*^	Odds ratio	*p*-value^*^
Controls (without CKD)	Per 10 mg/dl decrease		Per 10 mg/dl decrease		Per 10 kg decrease		Per 1 mg/dl decrease			
Model 1	1.01 (0.98, 1.04)	0.37	0.99 (0.93, 1.04)	0.68	1.00 (0.95, 1.05)	0.84	0.90 (0.55, 1.47)	0.70	1.05 (0.70, 1.58)	0.81
Model 2	0.98 (0.94, 1.02)	0.32	1.00 (0.93, 1.05)	0.93	0.89 (0.80, 1.01)	0.06	1.19 (0.68, 2.08)	0.53	1.11 (0.62, 1.96)	0.71
CKD (eGFR< 60 ml/min/1.73 m^2^)										
Model 1	1.05 (0.99, 1.12)	0.09	1.10 (0.97, 1.23)	0.08	1.06 (0.88, 1.28)	0.38	0.83 (0.21, 3.22)	0.77	2.45 (0.60, 9.94)	0.19
Model 2	0.96 (0.90, 1.01)	0.10	1.11 0.88, 1.41)	0.10	0.94 (0.61, 1.45)	0.80	1.52 (0.23, 10.00)	0.71	1.91 (0.48, 7.52)	0.33

Model 1 was univariable model

Model 2 was adjusted for age, sex, race, educational attainment, marital status, family income to poverty ratio, alcohol drinking, cigarette smoking status, diabetes, hypertension, obesity, history of cardiovascular disease, history of cancer and depression

^*^p-value was calculated by Sidak-Holm technique to adjust for multiple comparisons

## Discussion

Our study demonstrates that the prevalence of olfactory dysfunction and gustatory dysfunction among patients with CKD was high. After adjustment for confounders, CKD was significantly associated with olfactory dysfunction, but not gustatory dysfunction. The prevalence of olfactory dysfunction increased from CKD stage 3a to 5.

Our study adds to prior literature in several important ways. This is the largest analysis of olfactory and gustatory dysfunction using a nationally representative and well validated database in NHANES, which addresses several limitations of prior literature. One previous study by Frasnelli et al. [22] including 64 patients with CKD reported 56% had olfactory dysfunction. Three-fourths of the patients in this study had end-stage kidney disease (ESKD) on dialysis, which is likely has the most advanced olfactory/gustatory dysfunction, while nearly 90% of CKD in our study were CKD stage 3. Therefore, our analysis likely is a better representation of the broader CKD population. Another study by Nigwekar et al. [10] including 161 participants reported 41% of patients with CKD and 53% of patients with ESKD vs. 16% of controls having moderate to severe hyposmia. Moderate to severe hyposmia in this study defined as unable to correctly identify 26–30 or more of the 40 odors using UPSIT test. This definition corresponds to incorrectly identifying three or more of the eight odors using NHANES smell test as defined in our study. Although study by Nigwekar et al. included more CKD patients than study by Frasnelli et al., mean eGFR among CKD in this study was 31.3 ± 16 ml/min/1.73 m^2^ which still reflected a high proportion of severe CKD. Conversely, mean eGFR among CKD in our study was 45.4 ± 0.6 ml/min/1.73 m^2^ representing a broader CKD population. The higher prevalence of olfactory dysfunction in prior studies may be explained by the more severe CKD in participants in these studies that identified patients from outpatient nephrology and dialysis clinic. Our study thus highlights that even at much earlier stages of CKD, olfactory dysfunction may be an increasingly common problem relative to individuals with preserved kidney function. We also demonstrated an increasing trend in the prevalence of olfactory dysfunction as the CKD progressed which were not investigated in prior studies.

Moreover, our study reveals significant finding of reduced grip strength among patients having olfactory dysfunction with CKD. Olfactory dysfunction was shown to be associated with lower grip strength and sarcopenia mediated through reduced total body protein content in previous study [23]. However, olfactory dysfunction was not significantly associated with reduced grip strength among expanded CKD in our study. Expanded CKD included patients with albuminuria but with normal eGFR. It is possible that albuminuria with normal eGFR may not be associated with diminished grip strength as this is a very early stage of CKD. Other nutritional markers such as subjective global assessment (SGA) or protein catabolic rate were not assessed in our study.

Although the precise mechanism of olfactory dysfunction among those with kidney impairment remains unclear, there are several hypotheses. Firstly, vascular impairment including endothelial dysfunction, calcification of vessels and intimal thickening which is not uncommon among CKD population. Vascular impairment could lead to diminished cerebral blood flow and affect olfactory processing [24]. Secondly, oxidative stress and uremic toxins from impaired kidney function contribute to neuronal damage, decrease capacity and impair regeneration of olfactory epithelial cells, olfactory bulb and central processing of olfaction. Previous literature supported the role of uremia on olfactory dysfunction as there was an improvement in olfactory function among kidney transplant recipients and post dialysis. Additionally, it also suggested high plasticity and recovery capacity of the olfactory neurons after elimination of uremic toxins [1, 25, 26]. Interestingly, olfactory G protein-coupled receptors are also found in kidneys. Animal models demonstrate that these receptors help control blood pressure via renin secretion and help excrete glucose through urine via regulation of SGLT-1 expression in proximal tubule. However, ligands of these receptors in the kidneys remain mysterious and roles of these receptors in assisting with olfaction are still unknown [27–29].

Olfactory dysfunction is thought to be associated with malnutrition. Olfactory input play an important role in meal’s flavor, sensory appeal of food and food enjoyment [30]. The dysfunction in smell leads to food aversion, decreased appetite and inadequate food intake, which predisposes to poor nutritional status and malnutrition. Alternatively, poor nutritional status leads to vitamins and trace elements deficiencies which attribute to olfactory dysfunction [1, 26].

Our study pinpoints that olfactory dysfunction may exist even in patients with mild CKD, thus providing a potential opportunity for early and targeted interventions such as intranasal theophylline as described by prior study by Nigwekar et al. [10]. Theophylline is a nonselective phosphodiesterase inhibitor which increases intracellular cAMP and cGMP levels. As a result, the elevation in intracellular cAMP and cGMP levels activates vacuolar proton-pumping ATPase (V-ATPase) leading to an activation of epithelial ion channels which is thought to be a functional importance process in odor detection [31]. Interestingly, the activity of V-ATPase in renal proton secreting cells also increases [32]. The role of V-ATPase as a target for theophylline action requires further investigation. Early intervention with intranasal theophylline among CKD patients may help diminish incidence of olfactory dysfunction leading to better nutritional status.

In terms of gustatory dysfunction, Konstantinova et al. [9] reported that 53% of CKD patients had gustatory dysfunction. Nevertheless, this study subjectively collected gustatory dysfunction data from the response to “Can you feel any taste alteration?” which might not be accurate and tend to be overreported among those with comorbidities. Another study by McMahon et al .[33] reported the lower taste identification score among CKD stage 3–5 group after adjusting for age and sex but did not report prevalence of gustatory dysfunction. In this study, taste assessment was performed by asking participants to identify 5 primary tastes (sweet, sour, bitter, salty and umami) based upon 2 ml of solution representing each taste. This method was different from our study which used quinine whole-mouth test to assess for gustatory function. Quinine whole-mouth taste testing has been demonstrated to be a reasonable tool for overall taste functioning as described by previous studies [13, 34]. Our study showed only 13% of CKD had gustatory dysfunction and did not demonstrate significant association between gustatory dysfunction and CKD which were inconsistent with prior studies’ results. It is possible that quinine which binds to bitter taste receptor family 2 (T2Rs) and activates signaling cascade through phospholipase C β2 leading to inositol 1,4,5-triphosphate and diacylglycerol production might not be affected by CKD [35, 36]. While olfactory signaling pathway mostly activates adenylate cyclase 3 leading to cAMP production and the activity of cAMP may decreased among CKD patients [37, 38]. Alternatively, quinine might not be sensitive enough to reflect gustatory dysfunction among CKD mainly if the degree of kidney impairment is not severe. However, quinine has been shown to be a valid tool to represent overall gustatory function in general population [2, 13]. Previous literature proposed that high uremic toxins, zinc deficiency, reduced salivary flow rate and salivary pH changes might attribute to gustatory dysfunction among kidney impairment. However, these prior studies only assessed patients undergoing dialysis [39, 40].

Although previous studies demonstrated that serum zinc was found to be lower among CKD patients and may play a role in sensorial dysfunction including olfactory and gustatory function in this population, our study demonstrated no significant difference in serum zinc between participants with CKD and non CKD. Moreover, there were no significant differences in terms of serum zinc and the odds of either olfactory or gustatory dysfunction after adjusting for several confounders. It is possible that there were residual confounders in previous studies [11, 41, 42]. Possibly due to limited sample size in previous studies, none were adjusted for potential confounders such as age and comorbidities. Alternatively, the degree of severity in CKD included in our study was relatively mild compared to previous studies. Furthermore, there were only 4% of participants with zinc deficiency (< 60 μg/dL) in our study. Therefore, our study may not have enough sample size to represent those with zinc deficiency to demonstrate the association between serum zinc and olfactory and gustatory dysfunction. Future data is warranted to further analyze the role of zinc in olfactory and gustatory dysfunction among CKD.

Our study has several limitations. Firstly, our study included only among those aged > 40 as this was a group that NHANES included in smell and taste assessment. Secondly, the study design of NHANES is cross-sectional. Therefore, temporal relationship between olfactory or gustatory impairment and CKD as well as nutritional status could not be ascertained. Malnutrition may likely happen later on when olfactory dysfunction progresses. Thereby, our study may not capture the malnutrition in a cross-sectional analysis. In addition, we did not have data on ESKD undergoing hemodialysis or peritoneal dialysis or kidney transplant. Moreover, there were limited numbers of participants with severe kidney impairment that might represent CKD stage 4 or 5 which limited further analyses on the association between the degree of kidney impairment and olfactory or gustatory dysfunction. Finally, NHANES did not provide information on cognitive impairment among those aged less than 60 which might be a residual confounder. Apart from this, some aspects of nutritional status such as subjective global assessment (SGA) score were not assessed in NHANES 2013–2014 cycle. However, strengths in our study are worth mentioning. Our study is the largest study to include participants including CKD assessed for olfactory and gustatory function. This allows us to adjust for several potential confounders including socioeconomic status and several comorbidities with lower chance of overfitting. Moreover, our analysis incorporated analytic survey weights and design factors in the analysis to evaluate the prevalence of gustatory and olfactory dysfunction in general population with CKD.

In conclusion, CKD was associated with higher odds of olfactory but not gustatory dysfunction. Olfactory dysfunction was associated with lower grip strength among those with CKD. Early intervention to help identify and improve olfactory function among CKD requires further study, as it may provide a means to preserve muscle strength and improve nutritional status in this vulnerable population.

## Supplementary Information

**Additional file 1: Supplementary Table 1.** The association between expanded CKD and olfactory and gustatory dysfunction. **Supplementary Table 2a.** The association between olfactory dysfunction and nutritional markers among expanded CKD. **Supplementary Table 2b.** The association between gustatory dysfunction and nutritional markers among expanded CKD.

## Data Availability

All data generated or analyzed during this study are available in NHANES database and can be accessed through https://www.cdc.gov/nchs/nhanes/index.htm.
