# Supplementary material for: The association between olfactory and gustatory dysfunction and chronic kidney disease
Source: BMC Nephrol. 2022 Jan 18;23:36. doi: 10.1186/s12882-021-02659-6 (PMC8767746; doi:10.1186/s12882-021-02659-6)
Supplement: Supplementary file 1 — Additional file 1: Supplementary Table 1. The association between expanded CKD and olfactory and gustatory dysfunction. Supplementary Table 2a. The association between olfactory dysfunction and nutritional markers among expanded CKD. Supplementary Table 2b. The association between gustatory dysfunction and nutritional markers among expanded CKD. [file 12882_2021_2659_MOESM1_ESM.docx]

**Supplementary Materials**

**Supplementary Methods**

**Supplementary Table 1** The association between expanded CKD and olfactory and gustatory dysfunction

**Supplementary Table 2a** The association between olfactory dysfunction and nutritional markers among expanded CKD

**Supplementary Table 2b** The association between gustatory dysfunction and nutritional markers among expanded CKD

**Supplementary Methods**

**Sociodemographic Characteristics and other variables**

We defined diabetic individuals as those answering “yes” to the question “Other than during pregnancy, have you ever been told by a doctor or health professional that you have diabetes or sugar diabetes?” or having a hemoglobin A1C (HbA1C) more than 6.5%. Hypertension was defined as those answering “yes” to the question “Have you ever been told by a doctor or other health professional that you had hypertension, also called high blood pressure?” or having mean systolic/diastolic blood pressure greater than 130/85 mm Hg at the mobile examination center examination. Obesity was defined as those who had body mass index (BMI) ≥ 30 kg/m^2^. Participants were categorized as having a history of cardiovascular disease if they answered “yes” to any of the following the questions “Has a doctor or other health professional ever told you that you had coronary heart disease?”, or “Has a doctor or other health professional ever told you that you had a heart attack (also called myocardial infarction)?” or “Has a doctor or other health professional ever told you that you had a stroke?”. History of cancer was defined as those who answered “yes” to the question “Have you ever been told by a doctor or other health professional that you had cancer or a malignancy of any kind?”. Depression was assessed by the nine-item Patient Health Questionnaire scale (PHQ-9) and defined as those who had the score ≥ 10 reflected having moderate to severe depression (23).

**Supplementary Table 1** The association between impaired kidney function and olfactory and gustatory dysfunction

|  | Odds ratio of having olfactory dysfunction | | | | Odds ratio of having gustatory dysfunction | | | |
| --- | --- | --- | --- | --- | --- | --- | --- | --- |
|  | Crude | p-value | Adjusted^†^ | p-value | Crude | p-value | Adjusted^†^ | p-value |
| Expanded CKD (eGFR<60 ml/min/1.73 m^2^ or UACR >=30) | 2.04  (1.68, 2.49) | <0.001 | 1.29  (1.03, 1.60) | 0.04 | 1.40  (1.06, 1.84) | 0.02 | 1.41  (0.96, 2.09) | 0.08 |

^†^Multivariable logistic regression model was adjusted for age, sex, race, educational attainment, marital status, family income to poverty ratio, alcohol drinking, cigarette smoking status, diabetes, hypertension, obesity, history of cardiovascular disease, history of cancer and depression

**Supplementary Table 2a** The association between olfactory dysfunction and nutritional markers

|  | Total cholesterol | | LDL-cholesterol | | Grip strength | | Albumin | | Protein-Energy malnutrition | |
| --- | --- | --- | --- | --- | --- | --- | --- | --- | --- | --- |
|  | β-coefficient | p-value^*^ | β-coefficient | p-value^*^ | β-coefficient | p-value^*^ | β-coefficient | p-value^*^ | Odds ratio | p-value^*^ |
| Expanded CKD (eGFR<60 ml/min/1.73 m^2^ or UACR >=30) |  |  |  |  |  |  |  |  |  |  |
| Model 1 | -10.35  (-19.58, -1.12) | 0.09 | -4.95  (-19.11, 9.21) | 0.72 | -8.58  (-15.32, -1.83) | 0.04 | -0.10  (-0.17, -0.03) | 0.08 | 1.13  (0.56, 2.28) | 0.71 |
| Model 2 | -2.60  (-10.33, 5.12) | 0.86 | 0.82  (-7.42, 9.07) | 0.83 | -4.42  (-8.20, -0.63) | 0.11 | -0.10  (-0.18, -0.01) | 0.12 | 1.23  (0.45, 3.34) | 0.89 |

Model 1 was univariable model

Model 2 was adjusted for age, sex, race, educational attainment, marital status, family income to poverty ratio, alcohol drinking, cigarette smoking status, diabetes, hypertension, obesity, history of cardiovascular disease, history of cancer and depression

^*^p-value was calculated by Sidak-Holm technique to adjust for multiple comparisons

**Supplementary Table 2b** The association between gustatory dysfunction and nutritional markers

|  | Total cholesterol | | LDL-cholesterol | | Grip strength | | Albumin | | Protein-Energy malnutrition | |
| --- | --- | --- | --- | --- | --- | --- | --- | --- | --- | --- |
|  | β-coefficient | p-value^*^ | β-coefficient | p-value^*^ | β-coefficient | p-value^*^ | β-coefficient | p-value^*^ | Odds ratio | p-value^*^ |
| Expanded CKD (eGFR<60 ml/min/1.73 m^2^ or UACR >=30) |  |  |  |  |  |  |  |  |  |  |
| Model 1 | 1.35  (-10.34, 13.03) | 0.81 | 6.38  (-2.74, 15.50) | 0.16 | -4.60  (-10.96, 1.76) | 0.14 | 0.002  (-0.11, 0.12) | 0.97 | 1.56  (0.50, 4.80) | 0.41 |
| Model 2 | -0.13  (-9.63, 9.36) | 0.98 | 2.21  (-5.94, 10.36) | 0.57 | -5.36  (-9.91, 0.82) | 0.12 | 0.01  (-0.10, 0.12) | 0.81 | 1.31  (0.35, 4.91) | 0.67 |

Model 1 was univariable model

Model 2 was adjusted for age, sex, race, educational attainment, marital status, family income to poverty ratio, alcohol drinking, cigarette smoking status, diabetes, hypertension, obesity, history of cardiovascular disease, history of cancer and depression

^*^p-value was calculated by Sidak-Holm technique to adjust for multiple comparisons
